# Draft Genome Sequence of *Phaeomoniella chlamydospora* Strain RR-HG1, a Grapevine Trunk Disease (Esca)-Related Member of the Ascomycota

**DOI:** 10.1128/genomeA.00098-14

**Published:** 2014-04-10

**Authors:** Livio Antonielli, Stéphane Compant, Joseph Strauss, Angela Sessitsch, Harald Berger

**Affiliations:** a Austrian Institute of Technology GmbH, Health and Environment Department, Bioresources Unit, Tulln, Austria; b Fungal Genetics and Genomics Unit, BOKU University and Austrian Institute of Technology, Bioresources and Technologies Campus Tulln, Tulln/Donau, Austria

## Abstract

The Ascomycota species *Phaeomoniella chlamydospora*, in concert with other fungi, is a causal agent for grapevine trunk diseases. Here, we present the first draft of the *P. chlamydospora* genome sequence, which comprises 355 scaffolds, with a total length of 26.59 Mb and 7,279 predicted protein-coding genes.

## GENOME ANNOUNCEMENT

Grapevine trunk diseases, including young esca (Petri disease) and esca proper (black measles), have become some of the most important diseases wherever grapevines are cultivated. The typical symptoms of esca are discolored trunk and white rot, brown spots on fruits, and “tiger stripes” on leaves. Esca is caused or at least supported by the fungal species *Phaeoacremonium aleophilum* (*Togninia minima*), *Phaeomoniella chlamydospora*, and possibly other fungal species (1, 2). A genome draft of strain *P. aleophilum* UCR-PA7 has already been published (3).

The strain *P. chlamydospora* RR-HG1 was isolated from a grapevine trunk (*Vitis vinifera* cv. Rheinriesling) in Illmitz, Austria, in June 2013. The strain was cultivated on malt extract agar and tip purified, and DNA was isolated using the DNeasy plant minikit (Qiagen) for phylogenetic testing by internal transcribed spacer-large subunit (ITS-LSU)/rRNA gene sequencing. The sequencing was performed by GATC Biotech AG (Constance, Germany) using an Illumina MiSeq personal sequencer in paired-end 250-bp mode. A total of 14.51 million reads were received, representing 7,255.1 million bases in total. A read-quality check was performed using FastQC (http://www.bioinformatics.babraham.ac.uk/) and PrinSeq (4) and quality filtered using Trimmomatic (5) to obtain 11.74 million paired reads. SPAdes (6) was used for the scaffold assembly. Three hundred fifty-five scaffolds of >10 kb were obtained, with a mean coverage of 170× and a total length of 26.59 Mb. The quality of the genome assembly was assessed in QUAST (7) (*N*_50_, 100,274 bp; *N*_75_, 61,606 bp; length of shortest contig of those covering 50% of the assembly [L_50_], 80 bp; L_75_, 166 bp). A sequencing completeness of 96% was calculated using CEGMA (8). Gene annotation was performed using Augustus (9), previously trained on a gene structure created from CEGMA (8), and this method predicted 7,279 genes, the translated sequences of which were submitted to a BLASTp search against the nr database (NCBI).

Since *P. chlamydospora* is a plant pathogen, it can be expected to have a large number of genes putatively coding for plant cell wall-degrading enzymes (10). In our analysis, we predicted 37 beta-glucosidases (cellulose-degrading enzymes), 5 cutinases, 3 pectinases, 10 laccases, one lignin peroxidase, and 49 cytochrome P450 monooxygenases. It was already pointed out by Blanco-Ulate et al. (3) for *P. aleophilum* that these numbers are relatively low compared to those for other wood-degrading fungi.

Another important factor influencing the virulence of phytopathogens is their capacity to produce secondary metabolites (11–14). In RR-HG1, the numbers of genes predicted to be involved in secondary metabolite production, such as those encoding polyketide synthetases (PKS) and nonribosomal peptide synthetases (NRPS), are slightly lower (10 PKS and 12 NRPS genes) than those in other plant pathogens, e.g., *Fusarium graminearum*, with 15 PKS (15) and 15 NRPS (16) genes. This low diversity in secondary metabolite genes might indicate reduced pathogenicity and therefore be a reason why grapevines infected by *P. aleophilum* and *P. chlamydospora* frequently do not show foliar or fruit symptoms (17) or symptoms present only in grapevine plants older than 7 years.

### Nucleotide sequence accession numbers.

This whole-genome shotgun project has been deposited at DDBJ/EMBL/GenBank under the accession no. JACF00000000. The version described in this paper is version JACF01000000.

## References

[1] Surico G Mugnai L Marchi G 2006 Older and more recent observations on esca: a critical overview. Phytopathol. Mediterr. 45:68–86

[2] Bertsch C Ramírez-Suero M Magnin-Robert M Larignon P Chong J Abou-Mansour E Spagnolo A Clément C Fontaine F 2013 Grapevine trunk diseases: complex and still poorly understood. Plant Pathol. 62:243–265. 10.1111/j.1365-3059.2012.02674.x

[3] Blanco-Ulate B Rolshausen P Cantu D 2013 Draft genome sequence of the ascomycete *Phaeoacremonium aleophilum* strain UCR-PA7, a causal agent of the esca disease complex in grapevines. Genome Announc. 1(3):e00390-13. 10.1128/genomeA.00390-13 23814032 PMC3695428

[4] Schmieder R Edwards R 2011 Quality control and preprocessing of metagenomic datasets. Bioinformatics 27:863–864. 10.1093/bioinformatics/btr026 21278185 PMC3051327

[5] Lohse M Bolger AM Nagel A Fernie AR Lunn JE Stitt M Usadel B 2012 RobiNA: a user-friendly, integrated software solution for RNA-Seq-based transcriptomics. Nucleic Acids Res. 40:W622–W627. 10.1093/nar/gks540 22684630 PMC3394330

[6] Bankevich A Nurk S Antipov D Gurevich Dvorkin M Kulikov AS Lesin VM Nikolenko SI Pham S Prjibelski AD Pyshkin AV Sirotkin AV Vyahhi N Tesler G Alekseyev MA Pevzner PA 2012 SPAdes: a new genome assembly algorithm and its applications to single-cell sequencing. J. Comput. Biol. 19:455–477. 10.1089/cmb.2012.0021 22506599 PMC3342519

[7] Gurevich A Saveliev V Vyahhi N Tesler G 2013 QUAST: quality assessment tool for genome assemblies. Bioinformatics 29:1072–1075. 10.1093/bioinformatics/btt086 23422339 PMC3624806

[8] Parra G Bradnam K Ning Z Keane T Korf I 2009 Assessing the gene space in draft genomes. Nucleic Acids Res. 37:289–297. 10.1093/nar/gkn916 19042974 PMC2615622

[9] Stanke M Diekhans M Baertsch R Haussler D 2008 Using native and syntenically mapped cDNA alignments to improve *de novo* gene finding. Bioinformatics 24:637–644. 10.1093/bioinformatics/btn013 18218656

[10] Bruno G Sparapano L 2006 Effects of three esca-associated fungi on *Vitis vinifera* L. III. Enzymes produced by the pathogens and their role in fungus-to-plant or in fungus-to-fungus interactions. Physiol. Mol. Plant Pathol. 69:182–194. 10.1016/j.pmpp.2007.04.006

[11] Cumagun CJ Bowden RL Jurgenson JE Leslie JF Miedaner T 2004 Genetic mapping of pathogenicity and aggressiveness of *Gibberella zeae* (*Fusarium graminearum*) toward wheat. Phytopathology 94:520–526. 10.1094/PHYTO.2004.94.5.520 18943772

[12] Lee J Jurgenson JE Leslie JF Bowden RL 2008 Alignment of genetic and physical maps of *Gibberella zeae*. Appl. Environ. Microbiol. 74:2349–2359. 10.1128/AEM.01866-07 18263740 PMC2293157

[13] Tudzynski B 2005 Gibberellin biosynthesis in fungi: genes, enzymes, evolution, and impact on biotechnology. Appl. Microbiol. Biotechnol. 66:597–611. 10.1007/s00253-004-1805-1 15578178

[14] Pedras MS Montaut S 2003 Probing crucial metabolic pathways in fungal pathogens of crucifers: biotransformation of indole-3-acetaldoxime, 4-hydroxyphenylacetaldoxime, and their metabolites. Bioorg. Med. Chem. 11:3115–3120. 10.1016/S0968-0896(03)00241-4 12818674

[15] Gaffoor I Trail F 2006 Characterization of two polyketide synthase genes involved in zearalenone biosynthesis in *Gibberella zeae* characterization of two polyketide synthase genes involved in zearalenone biosynthesis in *Gibberella zeae*. Appl. Environ. Microbiol. 72:1793–1799. 10.1128/AEM.72.3.1793-1799.2006 16517624 PMC1393172

[16] Hansen FT Sørensen JL Giese H Sondergaard TE Frandsen RJ 2012 Quick guide to polyketide synthase and nonribosomal synthetase genes in *Fusarium*. Int. J. Food Microbiol. 155:128–136. 10.1016/j.ijfoodmicro.2012.01.018 22377171

[17] Hofstetter V Buyck B Croll D Viret O Couloux A Gindro K 2012 What if esca disease of grapevine were not a fungal disease? Fungal Divers. 54:51–67. 10.1007/s13225-012-0171-z

